# Strong Field-Induced Frequency Conversion of Laser Radiation in Plasma Plumes: Recent Achievements

**DOI:** 10.1155/2013/127670

**Published:** 2013-06-20

**Authors:** R. A. Ganeev

**Affiliations:** ^1^Saitama Medical University, Saitama 350-0495, Japan; ^2^Voronezh State University, Voronezh 394006, Russia

## Abstract

New findings in plasma harmonics studies using strong laser fields are reviewed. We discuss recent achievements in the growth of the efficiency of coherent extreme ultraviolet (XUV) radiation sources based on frequency conversion of the ultrashort pulses in the laser-produced plasmas, which allowed for the spectral and structural studies of matter through the high-order harmonic generation (HHG) spectroscopy. These studies showed that plasma HHG can open new opportunities in many unexpected areas of laser-matter interaction. Besides being considered as an alternative method for generation of coherent XUV radiation, it can be used as a powerful tool for various spectroscopic and analytical applications.

## 1. Introduction 

To promote the use of extreme ultraviolet (XUV) radiation, it seems therefore appropriate to advance laboratory scale sources to a higher application level. Many interesting experiments can be performed by high-order harmonic generation (HHG) based on femtosecond lasers. The strong-field process of HHG provides a source of coherent femtosecond and attosecond pulses in the extreme ultraviolet (XUV) with a wide variety of applications [1]. HHG is also a spectroscopic tool to extract structural and dynamical information on the emitting medium from the properties of the harmonic radiation (spectrum, phase, and polarization state). 

The harmonic generation sources easily cover the spectral range between 10 and 100 eV photon energy of harmonics, with few-cycles laser systems even up to several 100 eV. For practical applications of high-order harmonic sources, higher conversion efficiency and thus an increase in the photon flux and also of the maximum photon energy of the harmonic radiation would be beneficial. HHG itself can be used as a spectroscopic tool for analysis of the optical, nonlinear optical, and structural properties of the emitters of harmonic generation presently comprising on a few noble gases. The generation of high-order harmonics in laser-produced plasmas from various solid-state targets, being for this purpose a relatively new and largely unexplored medium, promises to yield these advances.

Below we briefly address the principles of HHG in isotropic media, such as gases and plasmas. If a single atom is tunnel ionized by an intense laser pulse, its outer-most electron will appear in the continuum at rest in the presence of an oscillating electric field. The phase of the field at the time at which the electron appears in the continuum is what will determine its destiny. When the ponderomotive potential becomes comparable or greater than ionization potential, it becomes easier to think of the process as nonperturbative. This is the most common way of looking at HHG. A semiclassical theory was first developed by Corkum [2] then a fully quantum mechanical model was presented by Lewenstein et al. [3].

**Figure 1:**
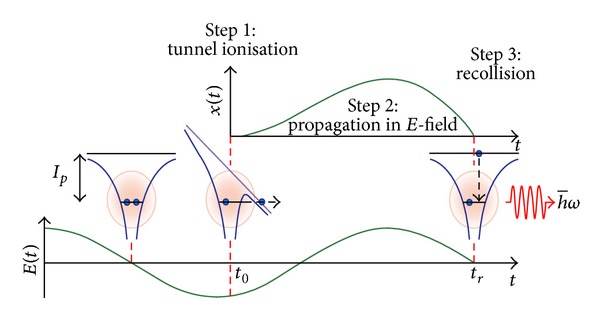
Three-step mechanism of high-order harmonic generation. Step 1: tunnel ionization, step 2: electron acceleration in the electromagnetic field of the laser wave, and step 3: recollision and recombination with the ion and the emission of harmonics [4].

The HHG can be described using the semiclassical three-step model (Figure 1 [4]). The first step is the ionization of the atom by the laser field, the second is the acceleration of the electron by the laser field, and the third is the recombination with its parent ion, which can lead to the emission of a harmonic photon [5]. The strong field approximation (SFA) can be used to simplify the calculations of the harmonic spectrum. The main approximations associated with it are that the bound electron does not feel the influence of the external electric field and that the continuum electron does not feel the influence of the ionic potential. This is justified by the fact that the ionization is dominated by tunneling, and it is therefore peaked around the time of maximum laser electric field. This means that the field is strong at the time when the electron appears in the continuum and the motion of the ionized electron is widely dominated by the laser field. When the laser field reaches zero, the electron is far from the ion core and this assumption is still valid. When the electron returns, its velocity is very high so the time the ionic potential has to affect the electron's trajectory is short and it is still a reasonable approximation to discard it. This is a semiclassical model in the way that the evolution of the electron in the continuum is treated classically and the ionization and recombination are treated quantum mechanically.

A few models were developed, which demonstrate a long plateau where the intensity stays around the same order of magnitude for many harmonic orders followed by a sharp cutoff. The SFA calculations locate the cutoff energy, *E*_co⁡_, to be at *E*_co⁡_ = *I*_*P*_ + 3.17*U*_*P*_, where *I*_*P*_ is the ionization potential of the atom, and *U*_*P*_ is the ponderomotive energy.

High-order harmonics have a number of interesting properties. They are a tunable table-top source of XUV/soft X-rays, synchronized with the driving laser and produced with the same repetition rate. The harmonic cutoff varies linearly with increasing laser intensity up until the saturation intensity where harmonic generation stops. The saturation intensity can be increased by changing the atomic species to lighter noble gases but these have a lower conversion efficiency and so there is a balance to be found depending on the photon energies required.

High harmonic generation strongly depends on the driving laser field and as a result the harmonics have similar temporal and spatial coherence properties. High harmonics are often generated with pulse durations shorter than those of the driving laser. This is due to phase matching and ionization. Often harmonics are only produced in a very small temporal window when the phase matching condition is met. Depletion of the generating media due to ionization also means that harmonic generation is mainly confined to the leading edge of the driving pulse. High harmonics are emitted colinearly with the driving laser and can have a very tight angular confinement, sometimes with less divergence than that of the fundamental field and near Gaussian beam profiles. 

Some interesting limits on the HHG process, which are explained by recollisional model, show that HHG will only occur if the driving laser field is linearly polarized. Ellipticity on the laser beam causes the returning electron to miss the parent nucleus. Quantum mechanically, the overlap of the returning electron wave packet with the nuclear wave packet is reduced. This has been observed experimentally, where the intensity of harmonics decreases rapidly with increasing ellipticity. Another effect, which limits the intensity of the driving laser, is the Lorentz force. At intensities above 10^16^ W cm^−2^, the magnetic component of the laser pulse, which is ignored in weak field optics, can become strong enough to deflect the returning electron. This will cause it to “miss” the parent nucleus and hence prevent HHG.

**Figure 2:**
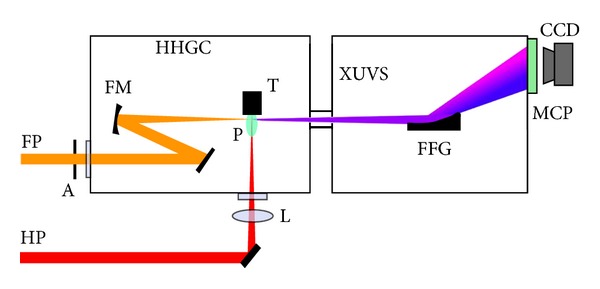
Experimental setup for harmonic generation in plasma plumes. FP: femtosecond probe pulse, HP: picosecond heating pulse, A: aperture, HHGC: high-order harmonic generation chamber, FM: focusing mirror, L: focusing lens, T: target, P: plasma, XUVS: extreme ultraviolet spectrometer, FFG: flat field grating, MCP: microchannel plate and phosphor screen detector, CCD: CCD camera [6].

Here we present a brief description of the experimental scheme commonly used for plasma harmonic generation. High-intensity pulses are typically obtained from the Ti:sapphire lasers. This radiation was used for frequency upconversion in the specially prepared plasmas. A portion of the uncompressed radiation of this laser (typically of the few tens to hundreds picoseconds pulse duration) was split from the beam line prior to the laser compressor stage and was focused into the vacuum chamber to heat the ablating target and create a plasma on its surface (Figure 2 [6]). The delay between plasma initiation and femtosecond pulse propagation was established in the range of few tens of nanoseconds. 

The femtosecond pulses propagating in a direction orthogonal to that of the heating pulse were focused into the laser plasma using the reflective mirrors. The position of the focus with respect to the plasma area was chosen to maximize the harmonic signal, and the intensity of femtosecond pulses at the plasma area at these conditions was estimated to be in the range of 10^14^–10^15^ W cm^−2^. The harmonics were analyzed using the XUV spectrometers. The details of these setups and registration systems are presented in [6, 7] and will also be presented in this review. 

Now, once we briefly discussed the basics of harmonics generation in isotropic media and described the experimental schemes, let us consider some results of the HHG in various plasmas produced by laser ablation. Whilst the first stage of these successful studies was entirely focused on the improvements of harmonic yield from plasma, at the current stage of knowledge of the high-order nonlinear optical processes in ablation plume one can consider this method as a new tool for material science. Thus the search of the dual role of plasma HHG as a method for efficient coherent XUV light generation and of materials probing is a milestone of further developments in this field. Below, we show new trends emerged during recent years, which demonstrate the attractiveness of this method. 

In this review, we discuss the realization of new ideas, which allowed further improvement of the HHG efficiency through harmonic generation in specially prepared plasmas and allowed the spectral and structural studies of matter through the plasma harmonic spectroscopy. We also present the current status of laser ablation induced high-order harmonic generation spectroscopy (LAIHOHGS) and show the perspectives in the developments of this filed. 

## 2. New Trends in Plasma HHG

Plasma HHG has become considerably mature during the last few years and continues to attract the growing attention of various laboratories worldwide. Currently, the experimental studies of plasma HHG are carried out in Japan, Canada, India, Uzbekistan, USA, Ireland, Germany, Korea, France, Spain, and the United Kingdom. Below, the most recent developments and some fresh approaches, experimental schemes, and ideas are described, which could considerably push this field toward the dramatic improvement of the output characteristics of harmonics and better understanding of the matter properties through the LAIHOHGS.

The intense HHG from plasma that is created from different carbon targets has recently been demonstrated using 10 Hz pulse repetition rate laser [8]. A high-order harmonic energy in the multimicrojoule range for each harmonic order from the 11th to the 17th harmonic was obtained. It was concluded, by analyzing the target morphology and the plasma composition, that the intense harmonics from the bulk carbon targets originate from the nanoparticles produced during ablation of the carbon-contained target. It was shown earlier that nanoparticles and films of C_60_ would generate harmonics that are more intense than those obtained from solid targets [9]. The disadvantage of using nanoparticle and film targets is the instability of the harmonics, which considerably vary from shot to shot and even disappear after a few laser shots if the target is not moved. On the contrary, in [8] it was found that carbon bulk targets can generate harmonics, comparable to those from the nanoparticle or C_60_ plasma targets.

The scanning electron microscope (SEM) image of plasma debris from a carbon target revealed that the plasma plumes contain nanoparticles with sizes varying between 100 nm and 300 nm. It was therefore suggested that, during the interaction of the heating pulse with the carbon target, nanoparticles are formed in the plasma and are then pumped by the fundamental pulse to induce the generation of harmonics. The harmonic intensity using bulk carbon target remained stable for several minutes, even without moving the target position. By creating the plasma during 5 min on the same place of the solid carbon target, the harmonic intensity does not decrease more than 10%, while the one from nanoparticles decreased more than 90% after a few seconds. It was also noted that, unlike most other solid targets, the highest harmonic order obtained with a carbon target does not exceed 21. According to the cut-off law defined by the three-step model, it was suggested that these harmonics are generated by neutral atoms, rather than ions as in the case of other solid targets.

Further developments of both the stability of carbon-containing plasma harmonics and their enhanced yield were reported in [10]. The importance of these parameters is defined by the applicability of converted radiation for various needs. Many efforts have been dedicated to the improvement of these characteristics during the long history of harmonic generation in gases. The multimicrojoule harmonics have been generated by energy scaling of gas HHG under highly optimized conditions [11, 12], which, however, have basically pushed gas HHG to its limits. Therefore, there is an urgent need to search for methods to generate even more intense harmonics and attosecond pulses. For these purposes, gas clusters [13] and plasma produced from nanoparticle targets [9, 14] can be used to increase the intensity of harmonics. In the former case, microjoule intense harmonics have been demonstrated in the range of 50–90 nm.

The exact mechanism of HHG from clusters is still debated. Various extensions of the three step model have been proposed [15–18]. The dominant channel generally considered is ionization and recombination to the same atom (atom-to-itself). Since clusters are dense media, there is also a possibility of recombination to neighboring ions [16, 17]. This atom-to-neighbor emission can produce incoherent radiation due to a lack of phase locking between the two atomic wave functions [18]. Another contribution to harmonic emission may come from a wave function partially delocalized over the whole cluster, from which electrons tunnel out of and to which they recombine coherently (cluster-to-itself), as it was discussed in recent studies of gas cluster HHG [19].

Nanoparticle targets have the problem of a rapid decrease in the HHG intensity with consecutive shots due to degradation of targets, which prevents them from being used in applications, especially in the case of high pulse repetition rate lasers. These exotic targets are also not always available in abundance. A new approach has been reported recently, which showed that efficient and relatively stable high-order harmonics of 10 Hz laser sources could be generated from a target that is readily available in the household, pencil lead of a mechanical pencil [10]. The measurements of the harmonic energy generated from plasma produced from pencil lead and the comparison with harmonics produced from C_60_ particles, which has proven to be one of the most efficient media for plasma HHG, showed the advantages of harmonics from the former medium. The important advantage of using pencil lead target is the shot-to-shot stability of the harmonic intensity over a sufficiently large number of shots.

To understand the uniqueness of the pencil lead plasma, the researchers analyzed using an SEM the ablated material debris deposited on silicon substrates that were placed close to the ablation plume. Those SEM images revealed that the plasma created from a pencil lead target contains nanoparticles whose mean size was in the range of 200 nm. They therefore suspected that due to the ablation of the pencil lead target by the heating pulse, the nanoparticles are formed on the target surface and remained in the plasma, which in turn led to generation of the intense harmonics.

From the experimental observations of stronger harmonics than in case of fullerene plasma and the morphology of plasma debris, the authors of [8, 10] inferred that the origin of the extremely strong harmonics from pencil lead and carbon plasmas is similar to those described for nanoparticle targets. The presence of nanoparticles in the plasma deposition and low cutoff suggests that neutral atoms of nanoparticles are the main source of intense harmonics from the pencil plasma. An explanation for intense harmonic generation from nanoparticles could be the higher concentration of neutral atoms due to the presence of nanoparticles. Unlike single atoms and ions, whose density quickly decreases due to plasma expansion, the nanoparticles retain densities that are close to its solid state. Combined with the higher harmonic efficiency of neutral atoms compared with their ions, the neutral atoms within the nanoparticle could generate high-order harmonics efficiently. The authors of [8, 10] estimated a conversion efficiency of ~10^−4^ for the harmonics in the plateau range.

The important issue of HHG from plasmas is related to the characteristics of generated harmonics. Whereas the conversion efficiency issue has been taken seriously during recent developments of this technique, which led to a considerable enhancement of harmonic pulse energy, no temporal characterization of plasma harmonic pulses has been performed up to recent time. This is a crucial element for applications of a new source of coherent XUV radiation. It should not be taken for granted that this harmonic emission has a nice attosecond structure. Indeed, the generation in plasma induces many sources of distortion: the higher electron densities and gradients will affect the generation of harmonics through phase mismatching and may result in distortion of both the harmonic spatial phase front and spectral phase. Furthermore, the temporal characterization itself raises problems, such as the probe beam distortions, target deterioration, and instability of harmonic intensity.

The first measurements of the attosecond emission generated from the under-dense plasma produced on a solid target were reported in [20]. They generated high-order harmonics of a femtosecond infrared (IR) Ti:sapphire laser focused in a weakly ionized chromium plasma. The characterization of the plasma attosecond emission was performed using the RABITT technique [21]. The measurement of the harmonic spectral amplitude and phase allows for direct access to the attosecond structure through a Fourier transform. The amplitude of each harmonic is easily given by the amplitude of the main photoelectron lines corrected for the ionization cross section.

The relative phase between neighboring harmonic orders is accessed through two-photon XUV + IR ionization of the target gas. When the IR beam is superimposed on the XUV beam in argon gas, sidebands appear in the photoelectron spectrum between the main lines. They correspond to two-photon transitions: absorption of a harmonic photon, *qω*_0_, accompanied by either absorption or stimulated emission of a laser photon, *ω*_0_. Since two coherent quantum paths lead to the same sideband, interferences occur, which result in an oscillation of the sideband amplitude as the delay, *τ*, between the IR and harmonic field is scanned with sub-IR-laser-cycle resolution. The phase of the oscillation is the phase difference between the two interfering channels. The phase difference, *φ*_*q*_ − *φ*_*q*+2_, between two consecutive harmonics can then be extracted, readily giving the group delay, also called emission time. From the phases, *φ*_*q*_, obtained by integrating the emission times and the amplitudes, *A*_*q*_, of the harmonic orders, one can reconstruct the temporal intensity profile. 

**Figure 3:**
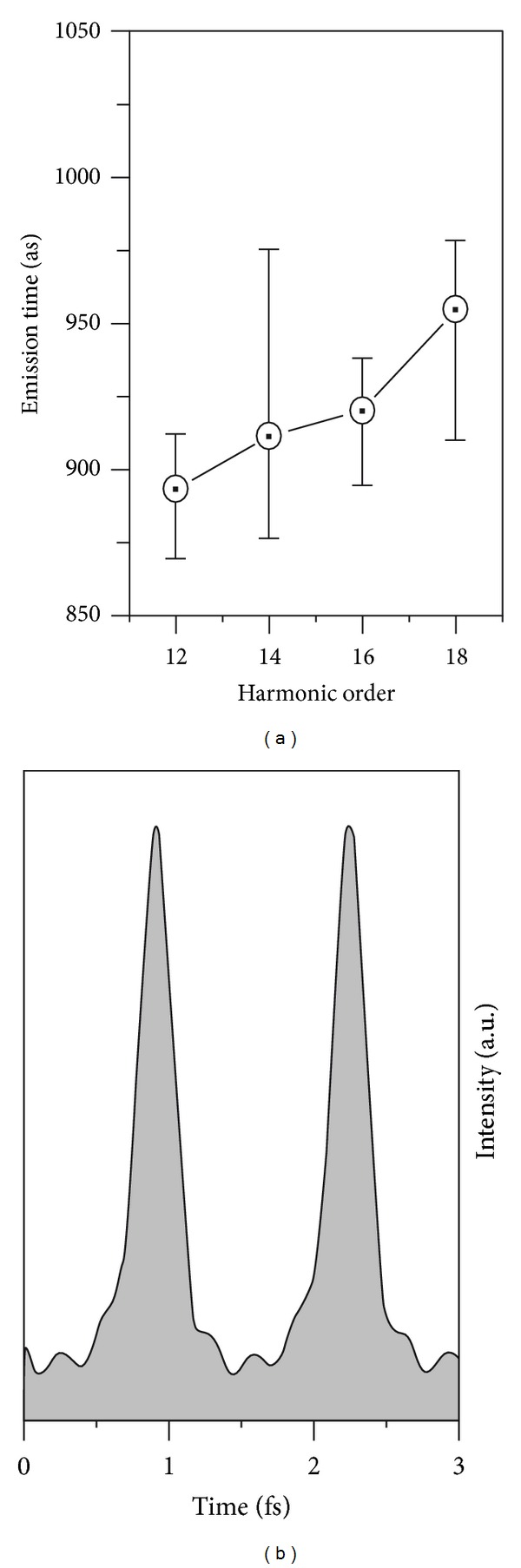
(a) Emission times, and (b) temporal profile corresponding to the 11th to the 19th harmonic orders [20].

The result for the measured five harmonic orders *q* = 11 to 19 is shown in Figure 3. The reconstructed temporal profile of the harmonic emission forms an attosecond pulse train, with each pulse of 300 as duration (full width at half maximum). Assuming that all five harmonics to be in phase, one can obtain the shortest pulses possible, that is, the Fourier-transform limited pulses. The corresponding duration is *τ* = 285 as. The measured duration of 300 as is thus only 1.05 times the Fourier transform limited duration.

Resonant enhancement of high harmonic generation can be obtained in plasmas containing ions with strong radiative transitions resonant with harmonic orders. In [22], the first temporal characterization of the attosecond emission from a tin plasma under near-resonant conditions for two different resonance detunings was performed. They have shown that the resonance considerably changes the relative phase of neighboring harmonics. These features were reproduced by simulations, allowing their interpretation in terms of the phase of the recombination dipole moment.

These studies give an affirmative answer to the practical question of whether resonance-enhanced HHG is indeed a source of intense ultrashort XUV pulses. The enhanced harmonic order has the same femtosecond duration as the nonresonant one. On the attosecond time scale, however, significant distortions of the phase of the near-resonant harmonic occur. They suggested the detuning from the resonance as an effective handle controlling the resonant harmonic phase. From a more fundamental viewpoint, previous studies of the HHG phase properties focused mainly on the phase accumulated by the quasi-free electron in the continuum, or on the recombination step as a probe of molecular structure and dynamics [23, 24]. This paper presents experimental evidence of the dramatic influence of the recombination step on the phase of resonant harmonics from an atomic target.

In the standard scheme of gas HHG, an ultrafast laser pump beam at intensities above 10^14^ W cm^−2^ is focused into a gas jet, generating high harmonics. The yield of such schemes is inevitably limited by dispersion in the medium. Across a distance equal to the coherence length, a phase mismatch of *π* grows and causes destructive interference between the pump and high harmonic beams. This process is one of the major limitations on the conversion efficiency of HHG. Quasi-phase matching (QPM) is a well-known approach for resolving this phase mismatch problem [25]. In QPM, the medium is modulated with a coherence length period so that the pump phase or harmonic emission is changed to prevent the destructive interference caused by the phase mismatch. For HHG in the XUV range and beyond, dispersion in the medium can be mostly attributed to free electrons generated using the laser ionization of the medium. Under this assumption, the coherence length (at 0.8 **μ**m wavelength) is given (in meters) by *L*_*c*_ ∝ 10^15^/*qN*_*e*_, where *N*_*e*_ is the free electron density (per cubic centimeter) and *q* is the harmonic number. Previously, QPM was realized by using multiple gas jets whose pressure and separation were properly controlled [26]. However, the realization of this technique is limited by geometrical constraints on the number and minimal separation of the jets.

**Figure 4:**
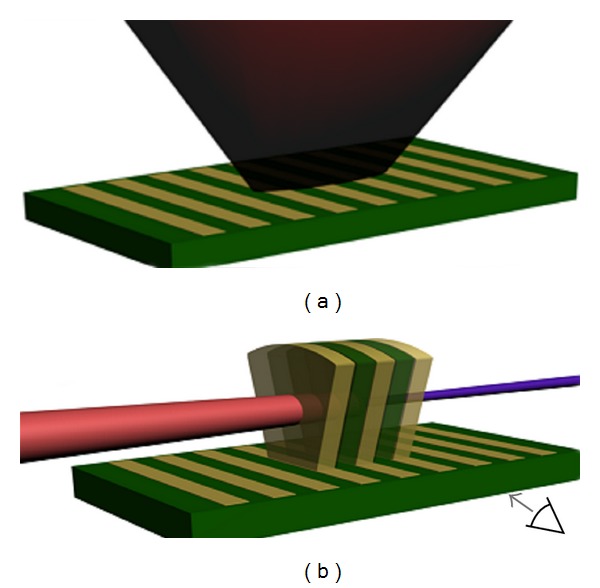
System schematics: (a) the lithographic pattern hit by a relatively low-intensity laser beam, and (b) the formed plasma jets in which the high intensity laser pump facilitates HHG [27].

It was proposed that the same procedure could be carried out for plasma HHG [27] using a simple method for fabricating numerous plasma jets tailored for the HHG, relieving technical restraints on the dimensions of the jets and their periodicity. In this scheme, the jets are produced by ablation of a microlithographic periodic stripe pattern (Figure 4). Cylindrical plasma jets formed by ablation extend the lithographic pattern into the space above the target, creating a row of narrow plasma jets of different material composition. The efficiency of HHG in plasma has been demonstrated to vary considerably with the atomic composition [28], and the periodic change in this efficiency enables QPM-HHG.

**Figure 5:**
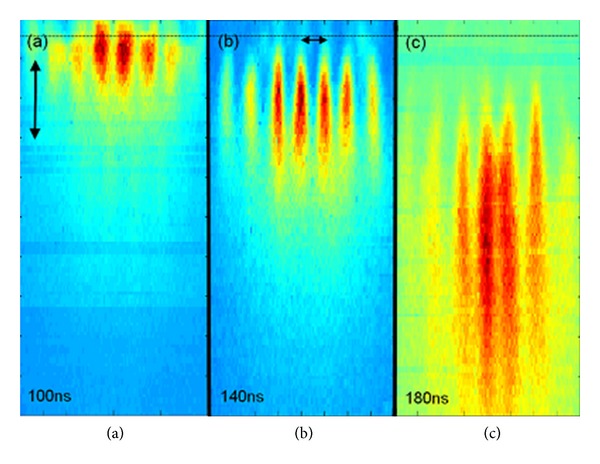
Monochromatic imaging of the plasma jets at different times using a 30 ns gate. The dashed line marks the target's surface, and the double arrows are 200 mm scale [27].

The results of [27] demonstrate a simple method for generation of periodic plasma structures by ablating a lithographic pattern. By passing a high-intensity laser pulse through such plasma patterns, suitable conditions for the QPM required for HHG can be created. These measurements suggest that such conditions exist between 140 and 180 ns after the initiation of plasma by the ablating laser pulse (Figure 5). Within this temporal window the plasma jets are several hundred microns wide and have relatively uniform temperature and relatively low electron density of ~10^17^ cm^−3^, whereas at later times the plasma structure begins to fade. Examining Figure 5 in depth shows that the modulation of the plasma density is significant, while the authors suggest that much finer periodicities suitable for generation of higher harmonics could be obtained by using finer lithography in preparation of the target. They have demonstrated the feasibility of a robust scheme for tailoring plasma structures with control over material composition, temperature, and density (both of free electrons and neutrals), through the ablation of specifically prepared lithographic targets, which can support the quasi-phase matched HHG.

## 3. Stable Generation of High-Order Harmonics of Femtosecond Laser Radiation from Laser Produced Plasma Plumes at 1 kHz Pulse Repetition Rate

Almost all HHG studies from weakly ionized plasmas produced during laser ablation of various solid targets were carried out using the 10 Hz pulse repetition rate lasers [29, 30]. Up to now, only few studies of HHG from plasmas have been carried out on static targets using 1 kHz class lasers [31–33]. The ablation process at 1 kHz pulse repetition rate causes a considerable change of the surface properties of the target due to the melting, which deteriorates the plasma plume conditions during laser ablation. Surface heating and melting of a static target result in an unstable harmonic signal so that movement of the target surface is required to maintain a reasonable stability. The demand in finding the optimal way for improving the plasma harmonic stability at 1 kHz pulse repetition rate is high due to recent observations of the advanced properties of plasma harmonics over gas harmonics [6, 8, 10]. In particular, in [8], the plasma HHG conversion efficiency was measured one order of magnitude stronger compared with gas HHG efficiency. Analogous features were reported in [6]. 

The obstacle of all plasma harmonic experiments during earlier studies was an insufficient stability of plasma parameters (density, ion and free electron concentrations, excitation conditions, etc.), which led to an instability of harmonic yield and fast decay of harmonic efficiency during irradiation of the same spot of ablating target. As it was mentioned, most of those early studies were performed using a 10 Hz class lasers. At this relatively low pulse repetition rate, the stability of harmonics deteriorated after a few hundred shots on the same spot of the surface and even quicker for powder-like materials (fullerenes, nanotubes, metal nanoparticles, various organic and nonorganic powders). One can note that laser ablation of the latter samples can be considered as an important tool for their structural studies using XUV nonlinear spectroscopy. The application of soft ablation allows the use of the same target for a much longer period than in the case of earlier studies of over-excited targets during laser ablation. Thus a search of a robust, easy-to-apply method for improving the harmonic stability in the case of plasma HHG could considerably advance a search of the fundamental (structural, orientational, etc.) properties of organic and inorganic atoms and molecules.

The earlier used approaches of a rotating disc geometry [34–36] for the movement of targets during ablation are not suitable since the distance between driving femtosecond beam and target surface should be maintained minimal (of order of 100 *μ*m), while the Rayleigh length of the driving beam is maintained at the range of few millimeters. Below we discuss a new method using a motorized rotating rod specifically prepared for the HHG from plasma plumes using high pulse repetition rate lasers and demonstrate that this target significantly improves the stability of high-order harmonics [37].

Those studies were performed using two laser pulses: one to produce the plasma plume and the second to drive the HHG within it. The first (heating) pulse was created by splitting off a portion (200 **μ**J) of the uncompressed 8 ps laser pulse from a 1 kHz Ti:sapphire chirped pulse amplification laser. The remaining pulse was compressed in a prism compressor and then further compressed using a hollow core fiber and chirped mirrors, resulting in 250 **μ**J, 3.5 fs pulses. The driving (probe) pulse was delayed with respect to the heating pulse by 40 ns to give the plasma the time to expand away from the target surface to allow the probe pulse to pass through the plasma without being clipped by the target. 

Target rotation apparatus consisted of three linear stages driven by stepper motors along three axes. The target was attached to an axis of the fourth motor, which provided rotation with a variable speed (from a few rotations per minute (rpm) up to 300 rpm). Rotating the target was sufficient to achieve stable harmonic radiation and an additional vertical movement was not required, though this capability might be useful for future plasma HHG experiments. As the setup requires the target to be positioned very close to the driving beam, it was of paramount importance that the target was carefully aligned to the axis of rotation. Any movement of the target surface due to eccentricity in the radial direction from the driving beam axis would result in an oscillation of the harmonic signal due to variation of the plasma density seen by the driving beam or, in the extreme case, clipping of the laser beam. 

**Figure 6:**
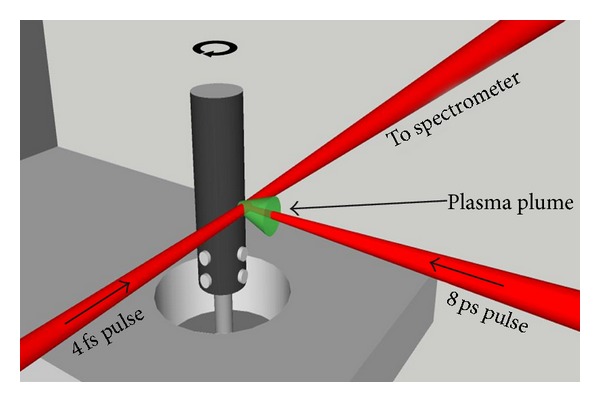
Schematic of the rotating target and HHG configuration [37].

The target (cylindrical rode with diameter of 10 mm and length of 30 mm) was positioned as shown in Figure 6, with the probe pulse propagating 100–200 **μ**m above the target surface. The picosecond heating pulse was focused onto the surface of the rotating target. In order to efficiently produce high-order harmonics, the plasma must be weakly ionized [38]. To achieve this, the target was positioned slightly in front of the focus of the heating pulse using a 50 cm focusing lens, leading to an on-target intensity of ~1 × 10^10^ W cm^−2^. This also had the benefit of increasing the size of the plasma produced from ablating a larger area. The size of the focus at the target surface was measured to be *≈*500 **μ**m. The delayed probe pulse was focused through the plasma using a 40 cm spherical mirror. The HHG radiation was analyzed by an XUV spectrometer consisting of a flat-field grating and an imaging microchannel plate detector with phosphor screen imaged onto a CCD camera.

**Figure 7:**
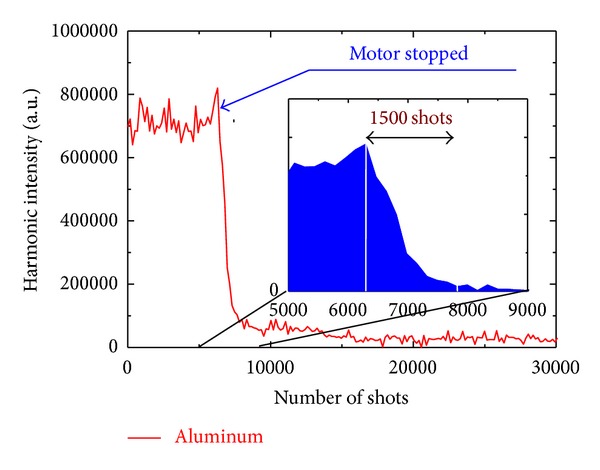
Decay of the harmonics from aluminum plasma after stopping the rotation of the motor. The harmonics were integrated over 40–80 nm spectral range [37].

**Figure 8:**
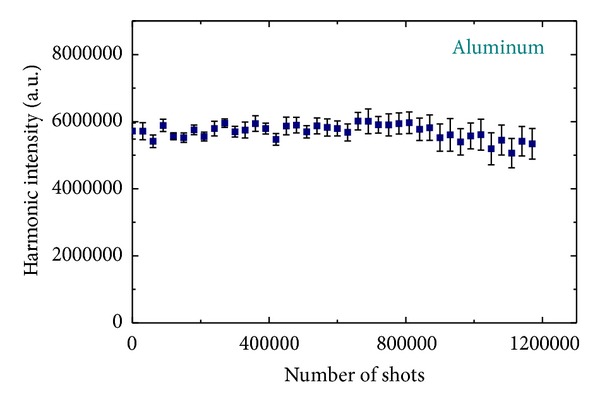
Stability of integrated harmonic signal from the aluminum plasma in the case of rotating target [37].

Figure 7 shows that there is a drastic change in the harmonic signal (integrated over the spectral range of 40–80 nm) when the rotation of the aluminum target is stopped. There is a sharp intensity decrease of more than one order of magnitude over only one thousand shots (or just after one second of ablation using 1 kHz laser). The benefits of the rotating rod are clearly shown in Figure 8 where stable harmonic generation was achieved from the plasma produced on an aluminum target for over 1 million laser shots. Stable harmonics were achieved in a broad range of the speeds of rotation (from 10 rpm and faster). The target rotational speed and the size of the ablation focus imply that the same area of target was undoubtedly used repeatedly for consecutive rotations over the 20 minute duration of experiments. This could result in thermal damage issues with this high pulse repetition rate. 

It is possible that once the fixed surface is melted the force from a following laser shot and plasma creation could expel some of the liquid target from the ablation area, which would not cause the plasma to be emitted in a direction normal to the surface. These effects are considerably diminished once the target starts to rotate. During rotation, the previously ablated area cools down such that, during the next set of ablation on this spot, the plasma formation occurs at approximately same conditions. To prove that the ablated area cools down with rotation, the target was rotated at different speeds (from 10 to 300 rpm) and no difference in stability of harmonic yield was found. These observations point out the importance of the periodic change of the ablation zone. This also confirms a suggestion that the cooling of the ablation area leads to stable plasma generation. 

Characteristics of plasma (density and ionization state) are the most important parameters to achieve and maintain stable HHG efficiency during an extended period of illumination. The calculations [29] have shown that, in the case of carbon plasma, the concentration of particles in the area of femtosecond laser-plasma interaction at optimal delay between heating and probe pulses (~40 ns) is ~2 × 10^18^ cm^−3^. The solid surface was considered as the one unheated before the laser ablation. Indeed, after one round of rotation (e.g., after 0.2–2 s), the plasma disappears, the ablated spot cools down, and the next laser shot on the same spot can be considered as a shot on the almost “fresh” surface. Contrary, in the case of a stationary target, the following shots continue the heating of the same spot.

The novelty of this approach includes the observation of advanced properties of plasma HHG even at extremely small energies of the heating pulses. The efficiency of plasma HHG depends on the possibility to create “optimal” plasma. This can be done using both multi-mJ pulses, as was shown in previous studies [29], and few hundred **μ**J pulses, as it was demonstrated in the reviewed work [37] and recently published studies [6]. The important point here is the intensity and fluence of the heating pulse on the target surface. The application of a higher energy heating pulse could create the conditions of “optimal” plasma over a longer distance, which could (or could not) increase the harmonic yield depending on the phase relations between the probe and harmonic waves. In addition, it can also lead to the overheating of the target at 1 kHz ablation. As it was already mentioned above, the rotating speed did not influence the stability of harmonics using 0.2 mJ heating pulses. The use of more energetic pulses at high repetition rate (i.e., of order of few mJ) may require additional optimization of the rotation target technique (e.g., by periodic up and down dragging of the rotating target).

## 4. High-Order Harmonic Generation in Graphite Plasma Plumes Using Ultrashort Laser Pulses

The characteristics of laser plasma play a crucial role in determining how efficiently high harmonics can be generated in the plasma plumes. An increase in the free electron density was likely to have been the limiting factor for the harmonic cut-off energy in early experiments with laser plasmas [34, 39, 40]. A search for appropriate target materials, which can provide favorable ablation plasmas for efficient HHG, has motivated the analysis of plasma characteristics at conditions of the high yield of harmonics. As it was mentioned above, recent studies have shown that carbon ablation plasmas are the promising media to satisfy the above requirements [6, 10].

Shot-to-shot stability of the harmonic signal is crucial for any application of the generated radiation and especially for the measurement of the pulse duration of converted XUV radiation. Such temporal measurements were reported in the case of HHG in chromium plasma ([20], see also Section 2). It was underlined that instability of the harmonic signal in their experiments using a 10 Hz pulse repetition rate laser was the main obstacle for an accurate measurement of the temporal structure of plasma harmonics. Beside its fundamental interest, HHG in plasma plumes could thus provide an intense source of femtosecond and attosecond pulses for various applications. 

Optical parametric amplifiers (OPAs) operating in the mid-infrared (MIR) range are promising tools for harmonic cut-off extension and attoscience experiments. The spectral cutoff energy of HHG obeys the scaling law *E*_*c*_ ~*Iλ* ^2^ [2], where *I* is the peak intensity of the probe field and *λ* its central wavelength, which allows one to extend the harmonic emission beyond the 100 eV range by using longer wavelength laser sources. Another advantage of mid-infrared optical parametric amplifiers (MIR OPAs) is their wavelength tunability, which allows one to tune the spectral position of harmonics towards the ionic transitions with strong oscillator strengths. This feature allows the observation of resonance-enhanced harmonics and broadens the range of plasma samples where this phenomenon could be realized compared with the case of ~800 nm lasers of essentially fixed wavelength [4]. Moreover, by using two-color HHG techniques, the application of MIR OPAs allows the study of complex molecules during their ablation and HHG using the tunable long-wavelength radiation. These features are interesting for spectroscopic applications of HHG in the MIR range [41, 42].

In the meantime, the use of MIR OPAs for HHG should lead to a reduced harmonic generation efficiency that scales as *λ* ^−5^ [43, 44]. It is of considerable interest to analyze the relative behavior of plasma harmonics in the cases of 800 nm and MIR lasers and thereby to find the conditions when the reduction of harmonic yield becomes not so dramatic due to some enhancement mechanisms, such as the presence of *in situ* produced nanoparticles, which increase the HHG conversion efficiency. It is worth noting that previous studies of plasma HHG in carbon plumes [8] have inferred, through analysis of plasma debris morphology, the formation of nanoparticles during laser ablation of carbon-contained targets.

Atomic carbon is a reactive species, which stabilizes in various multiatomic structures with different molecular configurations (allotropes). All the allotropic forms of carbon (graphite, diamond, and amorphous carbon) are solids under normal conditions, but graphite has the highest thermodynamic stability. Laser ablation of graphite has been intensively examined during the last ten years to define plasma conditions for the synthesis of carbon structures with unique properties. The physical characteristics of the plasma plume, such as concentration of atoms and clusters, directly affect the properties of the material being formed in the dynamic expansion of the ablated material. The successful synthesis of clusters is strongly dependent on the formation of atomic and molecular species with the required chemistry and aggregation ability. Thus, to select the optimal plasma conditions for HHG, a detailed understanding of the basic physical processes governing the ablation plume composition and reliable methods for controlling of the plume species are needed. The reasons mentioned above and the consideration of recent studies of HHG in carbon plasmas [8, 10], as well as recently reported comparisons of the HHG in graphite-ablated plasmas and argon gas [6, 45], have prompted to systematically analyze the plasma conditions for optimal HHG conversion efficiency in graphite plasmas [46].

High-intensity few-cycle pulses (760 nm central wavelength, 0.2 mJ, 3.5 fs pulse with 1 kHz repetition rate) were typically obtained from the Ti:sapphire laser after second stage of compression consisting of hollow fiber filled with neon and bunch of chirped mirrors [47]. The compressed pulses were characterized with a spatially encoded arrangement for direct electric field reconstruction by spectral shearing interferometry. This radiation was used for frequency up conversion in the specially prepared carbon plasma. A portion of the uncompressed radiation of this laser (central wavelength 780 nm, pulse energy 120 *μ*J, pulse duration 8 ps, and pulse repetition rate 1 kHz) was split from the beam line prior to the laser compressor stage and was focused into the vacuum chamber to heat the graphite target and create a plasma on its surface (Figure 2). These picosecond heating pulses were focused by a 400 mm focal length lens and created a plasma plume with a diameter of ~0.5 mm using an intensity on the target surface of *I*_ps_ = 2 × 10^10^ W cm^−2^. The delay between plasma initiation and femtosecond pulse propagation was fixed at 33 ns. As an alternative ablation, the 10 ns and 1064 nm pulses from a 10 Hz repetition rate Q-switched Nd:YAG laser were used that provided an intensity on the target surface of 1 × 10^9^ W cm^−2^. In that case, the delay between the 10 ns heating pulses and the 3.5 fs probe pulses was varied in the range of 10–60 ns to maximize the harmonic yield.

The 3.5 fs probe pulses propagating in a direction orthogonal to that of the heating pulse were focused into the laser plasma using a 400 mm focal length reflective mirror. The position of the focus with respect to the plasma area was chosen to maximize the harmonic signal, and the intensity of femtosecond pulses at the plasma area at these conditions was estimated to be *I*_fs_ = 6 × 10^14^ W cm^−2^. The 30 fs, 780 nm, and 2 mJ probe pulses from another Ti:sapphire laser operating at 1 kHz repetition rate and producing approximately the same intensity inside the laser plasma were also used for HHG. The details of this setup and registration system are presented in [6, 7]. 

In order to analyze the harmonic yield of the MIR source in the graphite-ablated plasma, an OPA pumped by the 30 fs Ti:sapphire laser was used. A beam splitter inserted before the laser compressor of this Ti:sapphire laser allowed to pick off 10% of the beam (780 nm, 1 mJ, 20 ps, and 1 kHz pulses) to generate a plasma plume on the graphite targets, with the remaining 90% being compressed to 30 fs (7 mJ) to pump a commercial OPA. The OPA was optimized for high conversion efficiency, beam quality, and duration of the converted pulses. To achieve high reproducibility of the generated pulses, all the amplification stages were driven to saturation. This device generated 35 fs pulses in the 1200–1600 nm range. The idler pulse covered the 1600–2200 nm range. The delay between the heating ablation pulse and MIR pulses from the OPA was set to 35 ns, as this delay was found to be optimal for the efficient generation of extended harmonics. 

Since the goal of these studies was to analyze the graphite ablation plasma characteristics at the conditions of efficient HHG of ultrashort laser pulses, this process was firstly optimized by achieving the maximum conversion efficiency and highest harmonic cutoff using the probe radiation from both the Ti:sapphire lasers with fixed wavelengths and the tunable OPA. Then the efforts were concentrated on the analysis of the “optimal” plasma plume using three techniques: optical emission spectroscopy of emitting plasma species in the visible, UV and XUV spectral ranges, scanning electron microscopy for inspection of the deposited plasma debris, and finally time-of-flight mass spectrometry for analysis of the ionic components of the plasma. 

**Figure 9:**
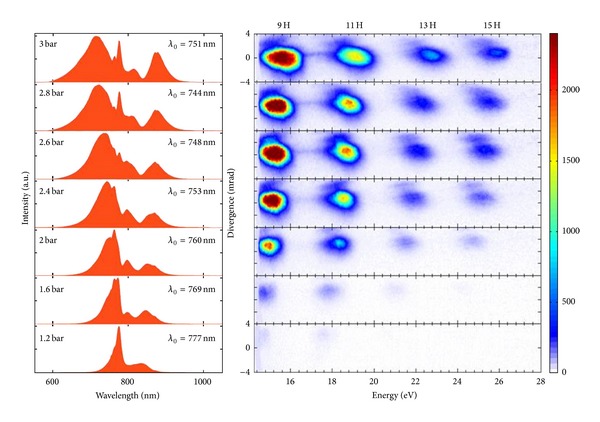
Carbon harmonic spectra as a function of neon pressure in the hollow fiber. The corresponding fundamental wave spectra measured in front of the vacuum chamber are presented on the left side. The plasma was created using the 10 ns pulses. *λ*_0_ is the central weighted wavelength of the spectral distribution. The color scale indicates the harmonic intensity [46].

To analyze the influence of the spectrotemporal characteristics of the probe radiation on the harmonic yield, the backing pressure of neon in the hollow fiber of second compressor was changed, which allowed the variation of pulse duration from 25 to 3.5 fs [48]. The dependence of the spectral and intensity characteristics of the harmonic images recorded by the CCD camera in the 15–25 eV range at different input pulse spectra and backing pressures of neon is shown in Figure 9. One can clearly see that, with the increase of backing pressure (from 1.2 to 3 bar), the harmonic intensity increases, while the harmonic wavelength spectrally shifts towards the blue. During these experiments, the driving pulse energy was held constant. 

An interesting feature of the carbon harmonic spectrum from the 10 ns pulse-induced plasma is that the spectral width is about 2-3 times broader than that of harmonics generated in other atom- and ion-rich plasmas at the same fluence and intensity of heating pulse, when using few-cycle pulses. For example, the full width at half maximum for medium-order harmonics was 1.5 nm in the case of graphite plasma, versus 0.4 nm for different metal (Ag, Al, and Cu) plasmas. The broader width of the harmonics can be explained by self-phase modulation and chirping of the fundamental radiation propagating through the carbon plasma. The presence of nanoparticles in the plasma plume may also contribute to bandwidth broadening of harmonics.

For practical applications of the coherent short-wave radiation generated in graphite plasma using a 1 kHz driving laser, it is necessary to analyze the stability of the plasma characteristics and the generated harmonics. A recently introduced new technique for maintaining a stable ablation plasma for harmonic generation using high pulse repetition rate lasers (>1 kHz) based on a cylindrical rotating metal target [37] was described in the previous section. The studies under discussion have shown that, in spite of different properties of metal and graphite targets, the rotating target allowed achieving stable HHG in both metal and graphite plasmas. The rotating graphite rod allows maintaining a relatively stable harmonic yield well above 1 × 10^6^ laser shots. Harmonics up to the 29th order were routinely observed in those studies using the 3.5 fs pulses.

**Figure 10:**
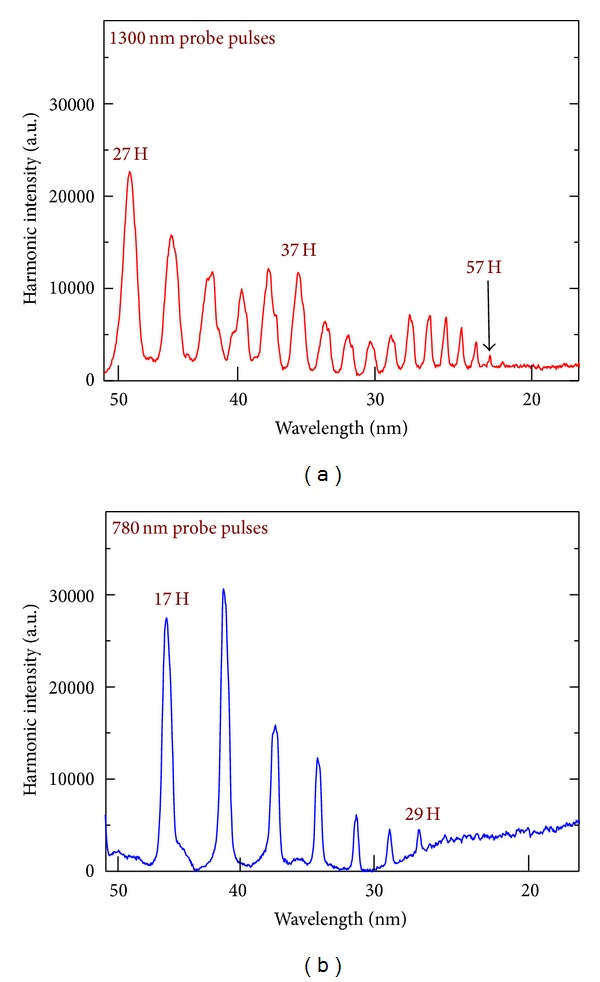
Carbon plasma harmonic spectra using the 1300 nm (a) and 780 nm (b) probe pulses. The energies of probe pulses were 0.2 mJ (a) and 0.54 mJ (b). Ablation was carried out using 20 ps, 780 nm, and 1 kHz laser pulses [46].

Figure 10(a) shows the harmonic spectrum generated in the case of 1300 nm probe pulses. Harmonics up to the 57th order were observed at the conditions of carbon plasma formation using the heating uncompressed 20 ps pulses from this laser. It is worth noting that application of less intense 1400 nm pulses available by tuning the OPA, while generating weaker harmonics, did not result in a higher harmonic cutoff than in the case of 1300 nm. This observation suggests that the harmonic generation occurred under saturated conditions, with the expectation of even stronger harmonics once the micro- and macroprocesses governing frequency conversion are optimized.

Harmonic spectra up to the 29th order in the case of 780 nm, 30 fs probe pulses are presented in Figure 10(b). By comparing with the spectra collected with the 1300 nm driving source (Figure 10(a)), one can clearly see the expected extension of harmonic cutoff in the case of the longer-wavelength driving source. The important peculiarities of these comparative studies are the broadband harmonic spectra in the case of 1300 nm laser and the similar yield of harmonics at the two driving wavelengths. Whilst the former feature depends on the bandwidth of the OPA output, the later observation requires additional consideration. The plasma harmonic yield from the MIR source did not follow the expected *I*_*h*_ ∝ *λ* ^−5^ rule. In fact, for the intensities of MIR and 780 nm pulses used (~ (2–4)×10^14^ W cm^−2^), the harmonic efficiency of the XUV radiation driven by MIR pulses was higher compared with the case of 780 nm pulses, while using lower energy of the former pulses (0.2 and 0.54 mJ, resp.). One can note that the *I*_*h*_ ∝ *λ* ^−5^ rule predicts a ~13-fold decrease of conversion efficiency for the MIR (1300 nm) pulses compared with the 780 nm pulses at equal probe pulse intensities.

An explanation for strong harmonic generation from nanoparticles compared with single atoms or ions could be the higher concentration of neutral atoms inevitably accompanying the presence of nanoparticles. The increase of electron recombination cross section for clusters with respect to atoms can also potentially enhance the HHG efficiency in nanoparticle-contained plasmas. Earlier studies of HHG from clustered gases [13, 42, 49, 50], as well as from the plasmas containing various nanoparticles (Ag, Au, BaTiO_3_, etc.) [4, 38], have proven these assumptions by demonstrating the enhanced HHG from clusters as compared with single atoms and ions. Further evidence of the cluster contribution to the enhancement of the harmonic generation comes from investigations of very intense laser ablation of a silver target [51], which gave the assumptions regarding the participation of *in situ* generated nanoparticles.

The observation of a strong extended harmonic plateau in the case of the 1300 nm probe radiation also suggests the involvement of clusters in the HHG process with MIR pulses. Assuming the expected decrease of harmonic intensity from single particle emitters with the growth of driving radiation wavelength (*I*_*h*_ *∝λ* ^−5^ [43, 44, 52, 53]), one can anticipate at least one order of magnitude decrease of harmonic yield from MIR pulses as compared with the harmonic yield obtained with 780 nm radiation at other equal conditions, in particular, pulse energy and duration. However, the experiment did not show a considerable difference between the intensities of harmonics originated from these two driving sources (Figure 10). The energy of the 1300 nm pulses in the plasma area (0.2 mJ) was lower than the Ti:sapphire pulse (0.54 mJ). This suggests the involvement of a mechanism, which compensates for the expected considerable decrease of harmonic efficiency for the longer-wavelength laser. The involvement of a clustered component of laser plasma in the process of frequency up conversion may arguably explain the observed inconsistence with the theoretical predictions of the *I*_*h*_ *∝λ* ^−5^ rule defined for atomic species [54, 55]. 

In principle, the enhancement of the harmonic spectrum from the carbon plume in the 15–26 eV range invokes the involvement of surface plasmon resonances of nanoparticles, analogously to the case of the fullerenes [56] in the range of their giant resonance in the vicinity of 20 eV. To prove this in the case of carbon plasma, one should provide evidence of giant absorption in the above range, but this has not been reported yet in the literature. The plasmonic properties of carbon nanoparticles can be responsible for the observed enhancement of carbon harmonics; however, their role requires additional study. Another option for explaining the high harmonic generation yield from the carbon plume is the indirect involvement of the clusters in HHG that, while not participating as harmonic emitters, could rather enhance the local field, analogously to recently reported studies using the gold nanostructures enhancing gas HHG [54, 55].

As it was mentioned, recent comparative studies of lower-order harmonic efficiency in argon gas and carbon plasmas have revealed stronger conversion efficiency in the carbon plasmas [45, 46]. In this subsection, we have discussed evidence of the superior properties of graphite ablation for HHG. Some arguments, which could explain the enhanced high-order harmonic yield from this medium, are as follows: (a) the graphite target allows easier generation of a relatively dense carbon plasma and the production of adequate phase-matching conditions for lower-order harmonic generation, (b) the first ionization potential of carbon is high enough to prevent the appearance of high concentration of free electrons, a condition that is not necessarily met in metal plasma plumes, (c) neutral carbon atoms dominate in the carbon plume at optimal conditions of HHG before the interaction with the femtosecond laser pulse, and (d) carbon species allow the formation of multiparticle clusters during laser ablation, which can enhance the HHG yield.

## 5. Isolated Subfemtosecond XUV Pulse Generation in Mn Plasma Ablation

In this section, we discuss HHG from transition metal plasmas. These are very promising targets in view of the giant resonances found in the photoionization cross sections. For example, the Mn^+^ cross section is ~40 Mb at 50 eV photon energy [57], whereas rare gas atoms have cross sections between 1 and 8 Mb at this photon energy [58]. Photorecombination, the third step in the recollision model, is the inverse process of photoionisation [59] and therefore HHG and photoionization must exhibit the same resonances. This has been confirmed not only by previous resonance-induced experiments with laser-produced transition metal plasmas but also in a recent study of HHG from xenon gas [60].

Resonance-induced enhancement of a single harmonic of the laser radiation allowed considerable improvement of harmonic efficiency in some specific XUV spectral ranges related with high oscillator strengths of ionic states of metals. This was confirmed in multiple studies following the initial observation of this phenomenon in indium plasma [61]. In particular, strong enhancement of a single harmonic was reported in Cr [62] and Mn [63] plasmas. The Mn plasma is of special interest since it showed the highest harmonic cut-off energy observed in plasma plumes (101st harmonic of Ti:sapphire laser [63]). In previous studies, multicycle (30 [63] and 140 fs [64]) laser pulses were employed and the generation of all harmonics in the plateau was observed together with a strongly enhanced harmonics in the vicinity of 50 eV.

Recent progress in the generation of few-cycle pulses allowed the observation of various new effects including the realization of isolated attosecond pulse generation in gas media [65–67]. In this connection it is interesting to analyze resonance-induced processes observed in an ablation plume using the shortest available probe laser pulses. Below, we present the analysis of the experiments on resonance enhancement in manganese plasmas using 3.5 fs pulses [68]. The most interesting feature observed in those experiments was a suppression of almost all neighboring harmonics in the vicinity of a resonantly enhanced single harmonic at the photon energy of ~50 eV. 

**Figure 11:**
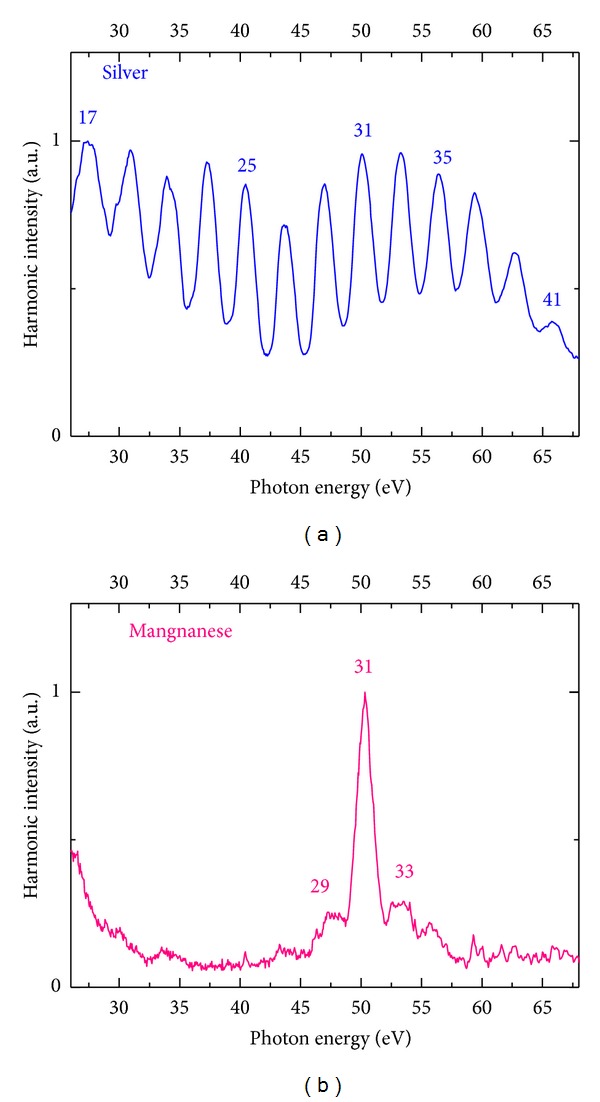
Harmonic spectra from the silver plasma (a) and manganese plasma (b) [68].

The experimental arrangements were analogous to those presented in the previous sections of this review. The harmonic spectrum in the case of propagation of the 3.5 fs pulses through the manganese plasma was strikingly different compared with other plasma samples (e.g., Ag plasma) analysed in separate experiments. While all other samples studied showed a relatively featureless harmonic spectra with extended cutoff (Figure 11(a) showing spectrum of the harmonics generating in the silver plasma), the Mn plasma allowed generation of a strong single harmonic substantially enhanced compared with neighboring ones (Figure 11(b)).

As it was already mentioned, the harmonic spectra from manganese plasmas for 30 fs and 140 fs pulses also showed enhanced harmonics around 50 eV. The assumption of the resonance nature of the enhancement of harmonics of the ~800 nm radiation of Ti:sapphire lasers in this spectral region is supported by the presence of a strong giant resonance in the vicinity of 50 eV confirmed by experimental [57, 69] and theoretical [70] studies. The enhancement of a single harmonic can be attributed to the broadband resonances of the ions of few metals, such as V, In, Cd, Cr, Cd, and Mn. These “giant” resonances have been experimentally confirmed in the literature [57, 69, 71] and discussed recently in a few theoretical studies [72–75]. 

**Figure 12:**
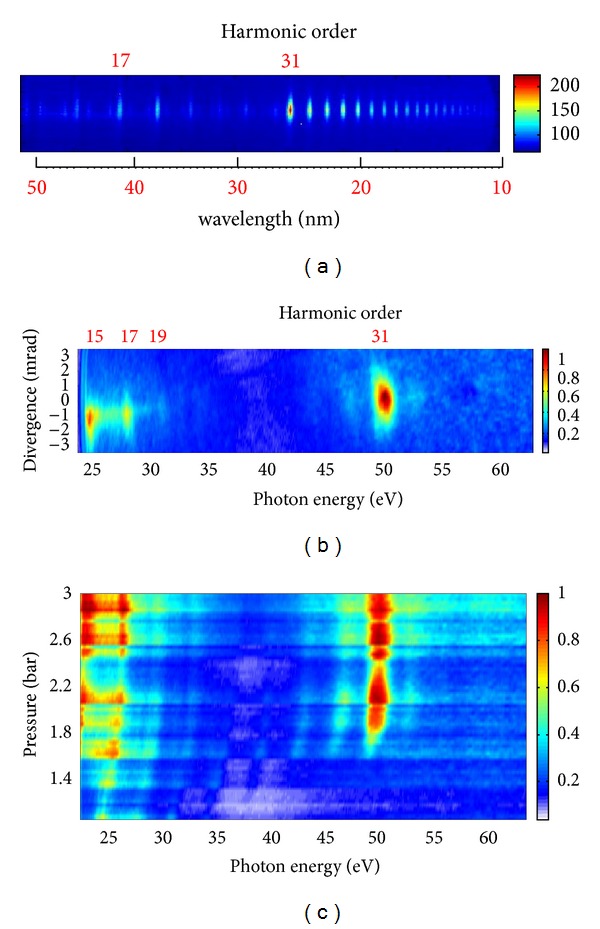
Raw images of harmonic spectra from manganese plasma in the case of (a) 40 fs and (b) 3.5 fs probe pulses obtained at the same intensity. (c) Raw images of harmonic spectra from Mn plasma at different pressures of neon in the hollow fiber obtained at the same energy of probe laser pulses [68].

However, in previous studies using multicycle probe pulses, the intensity of enhanced harmonics was only a few times higher compared with those neighboring harmonic orders. The same features were reproduced in the reviewed studies using 40 fs pulses from another Ti:sapphire laser at similar intensity inside the laser plasma (4 × 10^14^ W cm^−2^). The raw image of the harmonic spectrum presented in Figure 12(a) shows several enhanced harmonics starting from the 31st order followed by an extended second plateau. The extension of the harmonic cutoff exceeding the 71st order is attributed to the involvement of doubly charged Mn ions as the sources of HHG. This feature of Mn plasma harmonics has already been reported earlier [63]. Here also a typical image of an Mn harmonic spectrum in the case of 3.5 fs pulses is presented (Figure 12(b)). No second plateau, which was seen in the case of multicycle (40 fs) pulses, is observed for the few-cycle pulse. The most striking was the observation of a single, very strong, broadband (2.5 eV) 31st harmonic. Only two weak neighboring harmonics (around the strong emission) are seen in the 30–65 eV spectral range. The ratio between the intensities of the enhanced harmonic to the weak neighboring harmonics exceeded one order of magnitude. One can note that, at a lower intensity of the femtosecond pulse (<2 × 10^14^ W cm^−2^), this strong harmonic disappeared when using both multi- and few-cycle pulses. 

The distinctive structure of the harmonic spectra, both for 40 fs and 3.5 fs pulses, clearly points out the involvement of Mn resonances centered around 50-51 eV. The same can be said about the photoionization or photoabsorption characteristics of Mn^+^ plasma, which are due to the “giant” 3*p* → 3*d* resonance [57]. The laser polarization dependence of this emission was analyzed and it was found that the 50 eV radiation abruptly decreases with the change of the polarization state of the femtosecond driving pulses from linear to elliptical, which is a clear signature of the emission being due to high-order harmonic generation.

To analyze the effect of the spectrotemporal characteristics of the femtosecond radiation on the harmonic yield, the pressure of neon in the hollow fiber of second compressor was varied, thus changing the duration of the harmonic drive pulse [76]. The spectral and intensity variations of manganese harmonic spectra in the range of 22–62 eV as the functions of neon pressure in the hollow fiber are shown in Figure 12(c). One can clearly see that, with change of pressure (from 1 to 2.3 bar), the single 31st harmonic intensity varies from almost zero to its maximum high value. A blue shift of the harmonics is also evident. Further increase of neon pressure up to 3 bar, at which the experiments with the 3.5 fs pulses were carried out, did not change the harmonic distribution. 

**Figure 13:**
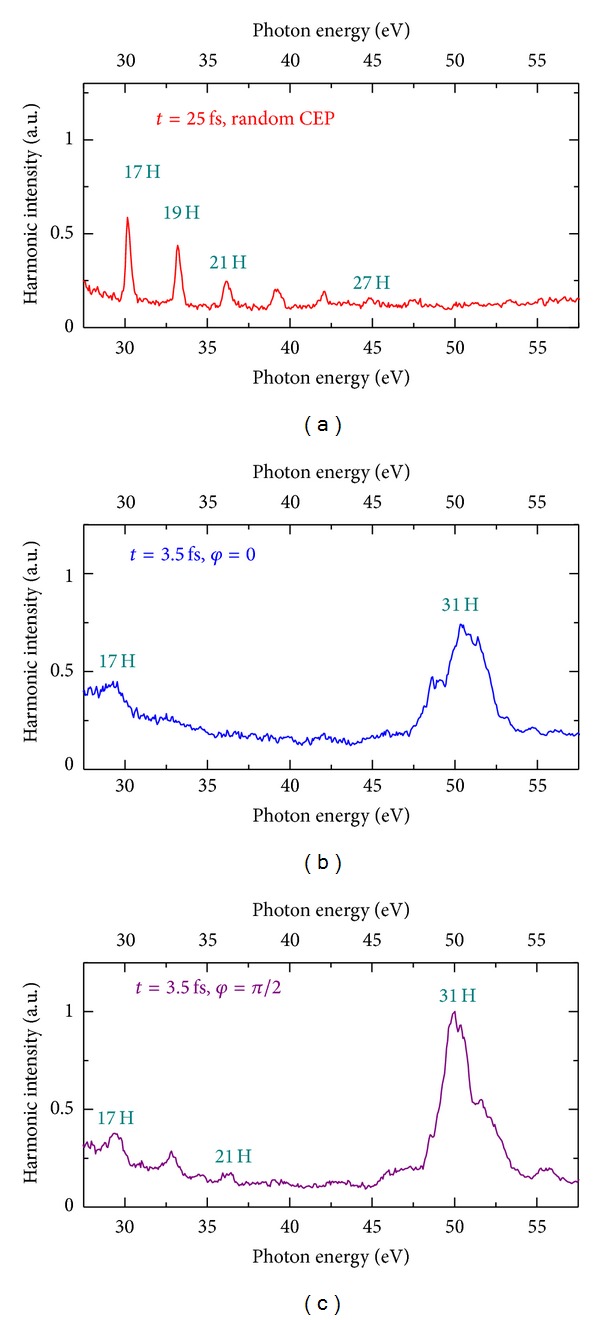
Experimental harmonic spectra generated from manganese plasma in the case of the absence of gas in the hollow fiber compressor (*t* = 25 fs) and random CEP (a), and at 3 bar pressure (*t* = 3.5 fs) at fixed CEP (*φ* = 0, (b); *φ* = *π*/2, (c)). *φ* denotes the carrier envelope phase [68].

The experiments described above were carried out without carrier envelope phase (CEP) stabilization (i.e., for random CEP values). The HHG experiments with Mn plasma using 3.5 fs pulses were also performed with stabilized CEP (e.g., at phases values of *φ* = 0 and *π*/2) and no considerable differences were found in that case (Figure 13), though some variation of harmonic distribution was observed for the lower order harmonics (compare Figures 13(b) and 13(c)). The spectral shapes of the 31st harmonic emission were quite similar for these two fixed values of CEP, while a considerable difference in harmonic spectra was maintained when comparing to longer pulse duration and lower intensity of the driving pulses. Figure 13 shows HHG measurements for 25 fs pulses (Figure 13(a)) and 3.5 fs pulses (Figures 13(b) and 13(c)) of the same energy. One can clearly see the absence of harmonic extension and resonance-induced HHG in the case of low-intensity, 25 fs pulses. 

The fact that a strong CEP dependence of the plasma harmonic spectra in the case of 3.5 fs pulses was not observed could also be attributed to the presence of a significant amount of free electrons in the manganese plasma, which might diminish the difference between the HHG spectra recorded for different values of CEP. The same can be said about other HHG experiments using silver and brass plasmas, which did not show significant differences in harmonic spectra when comparing few-cycle pulses with fixed and random CEP. In the meantime, comparative studies with gas media under similar experimental conditions were carried out and found a characteristic dependence of the HHG spectra on the CEP, which were commonly observed at such conditions. Thus the absence of the influence of the CEP on the harmonic pattern generated by few-cycle pulses from the ablation plumes appears to be a specific feature of plasma HHG. 

## 6. Perspectives of Plasma Harmonics

The quest for new plasma media that would favor the enhancement of an individual harmonic allows further enhancement of harmonic conversion efficiency. The production of a single high-intensity harmonic (rather than a group of harmonics of equal intensity in the plateau region) would open up the way to the practical application of these coherent short-wavelength radiation sources. Because resonantly enhanced harmonics have already been observed in several plasma media, there are strong grounds to believe that similar conditions will be discovered for other plasma formations. The generated harmonic wavelength may then be tuned to the transitions with high oscillator strength by wavelength tuning of the driving laser system, as well as by varying the chirp of the laser radiation. Application of ablated nanoparticles and clusters for HHG can also enhance the yield of harmonics in the XUV range. Further improvements in HHG conversion efficiency and harmonic extension require a systematic study of the influence of various plasma and laser parameters on ablation harmonics. Many new peculiarities of plasma harmonics emerged during the last few years allow expecting further extension of our knowledge of materials properties using this powerful took of nonlinear spectroscopy [77]. 

Whilst the first stage of these successful studies was entirely focused on the improvements of harmonic yield from plasma, at the current stage of knowledge of the high-order nonlinear optical processes in ablation plume one can consider this method as a new tool for materials science. Thus the search of the dual role of plasma HHG as a method for efficient coherent XUV light generation and of materials probing is a milestone of future studies. These studies are aimed at the enhancement of HHG efficiency from laser ablation produced on the surfaces of solid-state materials and applications of plasma harmonic studies for analysis of spectral and structural properties of materials.

Specifically, future studies are aimed on an analysis of the HHG spectroscopy conducted using a low-excited laser-produced plasma, which has already demonstrated several new approaches to the problem of increasing the HHG efficiency in comparison with the efficiency of conventional HHG in gases. At the same time, it is obvious that gas HHG exhibits much higher harmonic orders (in comparison with the plasma HHG obtained to date). Without making excessively optimistic predictions that the orders of plasma-generated harmonics will exceed the gas harmonic orders in the near future, we will focus primarily on the realization of new techniques for increasing the wavelength-converted radiation intensity with the use of plasma plumes, which may hardly be realized in conventional gas HHG. At the same time, one can explore various optical, nonlinear optical, and structural properties of materials using the LAIHOHGS. 

One can expect an improvement of plasma HHG due to the double excitation of laser-produced plasmas, optimization of the longitudinal harmonic generation scheme in the laser plume, optimization of nanostructured plasmas, use of multicomponent plasma plumes, formation of quasi-phase-matching conditions for the waves in complex extended plasmas, provision of a regime of waveguide pump propagation through the plasma medium, feasibility analysis of attosecond pulse generation in laser-produced plasma, use of mid-IR pulses for harmonic extension, stabilization of plasmas and harmonics characteristics using rotating and moving targets, search of the attosecond pulse generation by achieving the continuum in the harmonic emission near the cutoff, which allows for the first time the generation of ultrashort laser pulses in the plasma plumes, use of gating technique for shortening of harmonic pulses, and so forth. The joint implementation of new and old (application of the clusters with controllable and variable sizes, resonance-induced harmonic enhancement, two-color pump-induced enhancement of even harmonics, and search of the influence of multielectron dynamics of complex clusters, such as fullerenes and nanotubes, on the plasmon resonance-induced growth of few harmonics in the XUV range, etc.) methods and approaches in the case of plasma media allows making the advance to the state of the art within the project field, which will lead to further establishment of a new method of material science, LAIHOHGS. It follows from the above that investigations in this area of nonlinear optics are making rapid strides and may bring new success in the near future. 

It is worth noting that, currently, nonlinear spectroscopy involving gas HHG is trying to deal with such problems as (a) extension of the HHG spectroscopy-based attosecond structural imaging technique to image nuclear rearrangements induced by localized hole excitations, (b) application of strong-field ionization to create localized hole excitations and study their attosecond dynamics in polyatomic molecules, (c) search of selective imaging of hole dynamics induced by the removal of, for example, inner-valence electrons using the XUV initiated HHG technique, and (d) development of multidimensional HHG spectroscopy capable of following energy flow between different molecular modes over multiple femtosecond time scale. The proposed LAIHOHGS allows adding some important impulse to those studies, by using the peculiarities of plasma HHG. 

In the nearest future, the materials studies technique using the LAIHOHGS, which exploits the spectral and structural properties of various ablated solid-state materials using the propagation of short laser pulse through laser-produced plasma and generation of high-order harmonics, will be developed. The main goals of these studies will be a search of new schemes of high-order harmonic generation using the laser ablation, generation of femtosecond and attosecond XUV pulses through the HHG in plasma, enhancement of harmonic efficiency in these schemes, and the materials studies using the LAIHOHGS, which exploits the spectral and structural properties of various solid-state materials through their ablation and further propagation of short laser pulse through laser-produced plasma and generation of high-order harmonics. The important key step is putting, through ablation, solid molecules in gas phase at densities sufficient for HHG spectroscopy measurements. More concrete tasks and methodologies for achieving these goals are as follows.

1. New approaches in harmonic generation from the laser plumes: one has to carry out the analysis of the harmonic generation of ultrashort (multi- and few-cycle) laser pulses from the plasma produced on the surface of various targets at different conditions of experiment (driving laser wavelength and pulse duration, pulse repetition rate, excitation of plasma by nano-, pico-, and femtosecond pulses, variable plasma parameters, single atom, molecular, and cluster containing targets, etc.). Various new ideas for further amendments of this process will be examined. Among them are harmonic generation in plasma using two-color pump configuration in the case of commensurate and noncommensurate wavelength sources in the mid-infrared and ultraviolet ranges, search for the conditions of attosecond pulse trains generation using various gating techniques, measurements of pulse duration of the plasma harmonics generated by few-cycle pulses, studies of HHG from various clusters appearing *in situ* during laser ablation of targets, manipulation of plasma parameters by the second heating pulse propagating through the plasma simultaneously with driving femtosecond pulse, and analysis of phase matching and quasi-phase matching conditions for plasma HHG. The influence of molecular orientation on the harmonic output from molecules-containing plumes will be studied.
2. Analysis of plasma characteristics at “optimal” and “nonoptimal” conditions of HHG includes the studies of plasma composition using scanning electron microscopy, time-of-flight mass spectrometry of plasma components at the conditions of efficient HHG, development of stable plasma formation technique at high pulse repetition rate, and analysis of HHG at these conditions.
3. Development of ablation-induced HHG spectroscopy of various solid materials after their evaporation through laser ablation, particularly, the orientation-induced response of large ablated molecules and nanoparticles, and the time-resolved pump-probe analysis of complex plasmas containing various molecular structures.
4. Search of new schemes for resonantly enhanced harmonics: among the tasks are further studies of resonance enhancement, increase of the HHG efficiency, extension of the wavelength range for such enhancement, and use of two-color and mid-IR driving pulses for achieving the resonance conditions with the ionic transitions of various plasmas in different spectral ranges.
5. Development of various new approaches in plasma HHG, including the quasi-phase matching in extended plasma plumes, studies of the dynamics of aggregation and disintegration of clusters through their nonlinear optical response, comparative analysis of gas and plasma HHG at different conditions, joint application of gas and plasma HHG for the studies of gaseous and ablated species, creation of the conditions for single harmonic generation in the XUV range using few-cycle pulses, generation of continuum in the plasma harmonic spectra in different ranges of XUV using single and double gating techniques, and so forth.
6. The extension of the highest cutoff energy of generated harmonics using the interaction of intense laser radiation with doubly charged ions and analysis of harmonics extension in the case of longer wavelength (1200–2000 nm) driving radiation.
7. Harmonic generation from the laser plumes containing nanoparticles: here one can consider the influence of the surface plasmon resonances of clusters in plasma on the resonance-induced enhancement of harmonics, the analysis of plasmonic properties of carbon nanoparticles for enhancement of harmonics, and the studies of the indirect involvement of clusters in HHG, when they did not participate as the harmonic emitters, but rather enhance the local field, analogously to recently reported experiments and calculations using the gold nanostructures enhancing gas HHG.
8. Application of mid-infrared radiation to study the dynamics of nonlinear optical response of ablated solids compared with commonly used 800 nm class lasers for plasma HHG, including the studies of extended harmonics at comparable conversion efficiency with shorter wavelength laser sources, and a search of new opportunities in improvement of HHG conversion efficiency in the mid-IR range. The nonlinear optical interaction of ablated molecules with intense 800 nm and longer wavelength laser sources. In the latter case (mid-IR pulses), this interaction up-converts the frequency of the incident radiation by a factor of ~10^2^ yielding plasma harmonics of the incident radiation extending to the spectral region below 10 nm.
9. Separate topics of these studies will be the application of ablation plumes for attosecond science. Measurement of physical processes with a temporal resolution approaching 10^−16^ s has emerged in the last few years as one of the most exciting frontiers in physical science. Such measurements make accessible the ultrafast dynamics of correlated electronic motion that underpin the first moments of a wide range of physical and chemical processes, for example, in photochemical reactions, radiation damage in biomolecules and in converting light energy into chemical energy. This science demands the most advanced technology and in particular suitable light sources of exceptionally high bandwidth (>10 eV) to support the ultra-high temporal resolution. Presently, the primary technique to do this is high order harmonic generation in gases. The achievements in this field include generation of isolated attosecond pulses from atomic gases and use the time-energy encoding of the HHG spectrum to deduce subfemtosecond nuclear and electronic dynamics in the molecule. Nevertheless the capabilities for attosecond measurements are severely limited by several factors including (a) low photon yield in the generation of isolated attosecond pulses, and (b) the limited range of molecules that can be obtained in gas phase at densities sufficient for HHG.

The purpose of this research will be to enable various groups to fully develop world-leading capabilities in attosecond sciences using these newly identified plasma HHG spectroscopy methods. Although the work is challenging, one can expect that the present expertise in this field has the possibility to make a step-change advance in plasma harmonics induced attosecond science. The main objectives of this research are as follows:

- to test if, as expected from calculation, the resonance enhanced HHG in some ablation plumes leads to isolated subfemtosecond pulses using the attosecond streaking technique,
- To utilize the isolated subfemtosecond pulses from these metal plasmas in pump-probe measurements at surfaces and in molecules,
- To develop optimal conditions for ablation plumes of high density for HHG studies of intact ribonucleic acid and deoxyribonucleic acid bases (e.g., uracil and thymine) to allow the first steps for attosecond investigation of electron's dynamics in these molecules,

Currently, many scientific groups became interested in the development of laser ablation-induced plasma sources as the media for harmonic generation and as a tool for LAIHOHGS. A successful implementation of new ideas in this field requires the appropriate skill, which has been grown during recent years. All the above proposed goals and tasks are new and was not explored previously, excluding few approaches, which are currently under consideration in various laboratories worldwide. The novelty of the majority of the proposed ideas and the availability of resources allow expecting a successful realization of the above tasks, which further extend our knowledge in the field of materials science using the application of strong laser fields for nonlinear plasma spectroscopy.
